# The Westminster Conference

**Published:** 1921-08-06

**Authors:** 


### THE PUBLIC WELL-BEING.

### The Westminster Conference.

### AN ONLOOKER'S REFLECTIONS.

In the old days, when even general medical journals were few, and special ones non-existent, tuberculosis congresses and their reports were important things. The few special workers practically all met, and fraternised, and spoke, and compared notes. But of late years the importance has diminished. There is a huge special literature, there are text-books by the dozen; and the tuberculosis physician can, in his study, command the views of the rest of the civilised and progressive world. On almost every point facts have accumulated, until to try to attack the smallest one in a ten-minutes' speech—which, with a roomful of potential speakers and a two-hour-and-a-half meeting, is a liberal allowance—is felt by the sensible and unassertive man who really knows the subject to be absurd. It is the same change that has overtaken the formal lecture at a medical school. The students are present, but their thoughts are with their books, or the demonstration in ward or laboratory, or peradventure they are on the football field. The forward march of medicine has been such that it has outgrown communication by word of mouth. Both congress and lecture now, much more than of old, are functions, ceremonies—"gestures," as the cant phrase goes.

When structures are in a vestigial condition they are especially liable to disease; and all is not well with this last tuberculosis conference. When the preliminary announcement was submitted, some disagreement, both overt and covert, arose from the fact that Germany, Austria, and Russia were unrepresented. To the credit of some British tuberculosis workers be it said that it was felt to be unsatisfactory, to say the least, that while new ideas like the *x*-ray treatment for cancer at the West London Hospital are being imported straight from Germany, and whilst British and French workers (for example, Professor Calmette at the conference) cannot write good papers without plenty of reference to German and Austrian work, these countries should be boycotted. We believe that, finally, there was one Austrian delegate present. But to join in political strife, after peace has been declared, is an act upon which science, medical science above all, should turn its back. We are aware that war may leave bitterness, but then often individuals are never so friendly, from mere reaction, as just after a contest. What is at the bottom of the split, which invalidates this conference's international status, is something more than this. It is a feeling of triumphant virtue refusing to shake hands with defeated vice. Well, three or four years ago that was a view much more widely held than now; but what every person of common-sense wants is peace everywhere. If scientific workers would treat this matter as they do their own subject, and study a few of the original sources of both sides, now available if looked for, then the feeling of self-righteousness would everywhere yield, to be replaced by an even stronger one of hatred against the world-old "art of political lying."

There is an old saying that a bad beginning sometimes makes a good ending. We do not deny that Professor Calmette gave an interesting lecture. A high priest of bacteriology naturally views the problem of tuberculosis through a test-tube. But, as Dr. Ransome, who is happily still with us, long ago pointed out, the sociological aspect is at least as important as the pathological one. Practically, perhaps more so. Professor Calmette claims that to make the bacillus of Koch like the dodo we must isolate not only advanced cases, not only incipient ones, but also the ambulant "carrier," who may have a trifling sinus in the neck or even show no lesion at all. This, as our contemporary *The Lancet* tactfully intimated, would mean supervision of some twenty or thirty millions of people in these islands alone; people, we would add, not now in the best of tempers. Bacteriological demands have reached an *impasse*. Already the advanced case may have to wait a year for admission to hospital, and sometimes anticipates it by death. Happily in the past bacteriological claims have been known to recede with time and reflection. Professor Calmette is not quite accurate, by the way, in saying that only wild cattle are free from *perlsucht*, which domestication, like civilisation in man, causes to appear in them. Dr. Lankester, in his recent able study of tuberculosis in India, gives full particulars of a curious freedom from the disease of the domestic cattle of many parts of that country, in spite of bad conditions of life and much surrounding human tuberculosis. Such freedom even extends to a partial immunity to experimental inoculation, according to some experiments at the Parel Laboratory by Colonel Glen Liston.

Dr. Armand-Delille outlined the beneficent and common-sense measure of boarding out in country districts the as yet uninfected children of urban tuberculous parents. Will it be believed, however, that he claims that "practically it is the suppression of tuberculosis"? The obvious fallacy is that children from non-infected homes get tuberculosis too; and very commonly, as every tuberculosis officer knows. Besides, juvenile tuberculosis is not unknown in rural districts themselves. We fear that huge measures of social amelioration, which demand time for their fruition, are the pre-requisite, not mere quarantine measures, and it is bad tactics to extol so highly the latter, because Governmental authorities will use these high-pitched claims to fend off demands for more houses and cleaner milk. Indeed, there was a quiet suggestion of this attitude in speeches by politicians made at the beginning of the meeting. As a friendly onlooker, then, we urge more caution and less cocksureness.

